# High efficiency sorting and outgrowth for single-cell cloning of mammalian cell lines

**DOI:** 10.1007/s10529-022-03300-8

**Published:** 2022-09-08

**Authors:** Adonary Munoz, José M. Morachis

**Affiliations:** NanoCellect Biomedical Inc, https://nanocellect.com/

**Keywords:** Cell line development, Single-cell cloning

## Abstract

Single-cell selection and cloning is required for multiple bioprocessing and cell engineering workflows. Dispensing efficiency and outgrowth were optimized for multiple common suspension (CHO ES, Expi293F, and Jurkat) and adherent (MCF-7, A549, CHO-K1, and HEK293) cell lines. Single-cell sorting using a low pressure microfluidic cell sorter, the WOLF Cell Sorter, was compared with limiting dilution at 0.5 cells/well to demonstrate the increased efficiency of using flow cytometry selection of cells. In this work, there was an average single cell deposition on Day 0 of 89.1% across all the cell lines tested compared to 41.2% when using limiting dilution. After growth for 14 days, 66.7% of single-cell clones sorted with the WOLF Cell Sorter survived and only 23.8% when using limiting dilution. Using the WOLF Cell Sorter for cell line development results in higher viable single-cell colonies and the ability to select subpopulations of single-cells using multiple parameters.

## Introduction

Single-cell cloning (SCC) is an important step in generating monoclonal cell lines. It has numerous applications, such as therapeutic protein production, drug screening, gene therapy, and it can aid in the understanding of cell function and disease mechanisms (Soitu et al. 2019; Bode et al 2021). SCC applications require colonies that originate from a single cell to avoid discrepancies in product quality, inaccurate reproducibility of results, recombinant proteins instability, and poor outgrowth (Castan et al. 2018). These clones are traditionally validated by microscopy and is required by regulatory agencies for cell lines expressing therapeutics for commercial manufacture (Chen et al 2020). Liming dilution is a common method for SCC that relies on diluting cells to low concentrations in order to obtain a single cell per well based on Poisson distribution. However, limiting dilution is inherently inefficient and most wells end up with zero or more than one cell. The desired SCC method should be capable of discriminating cells of interest from a heterogeneous population and be able to deposit a single cell per well for the colony to have single-cell origin (Castan et al. 2018). Isolation of single-cells is typically accomplished using fluorescence activated cell sorting (FACS), micro-aspiration methods, or laser-capture microdissection. FACS is by far the most widely used technique due to its ability to sort based on one or more parameters in a high-throughput manner. The downside of commercially available “jet-in-air” cell sorters is their complexity, cost, and issues associated with high pressure sorting (Llufrio et al. 2018; Box et al. 2020). FACS combined with microfluidics is a modern option for isolating target cells without the downsides of traditional cell sorters.

In this work, the WOLF Cell Sorter with the N1 single Cell Dispenser (NanoCellect Biomedical Inc, San Diego, USA) was used to perform SCC of eight common adherent and suspension cell lines and compared with limiting dilution. Growth media was optimized and cell survival was measured up to 14 days. Finally, to mimic selection of engineered cells, a transduced cell line expressing varying amounts of green fluorescent protein (GFP) was sorted to create high expressing single-cell clones.

## Materials and methods

### Single cell deposition with dragon green beads

Single cell deposition was validated with 15 µm Dragon Green Beads (Bangs Laboratories Inc, Indiana, USA). The samples were prepared in 1X phosphate-buffered saline (PBS) (Genesee Scientific, San Diego, CA) and diluted to a final concentration of 1.0 × 10^5^ beads/mL that were sorted at 1 cell per well using the WOLF. Cells were sorted into three 96-well plates that were pre-filled with 200 µL of 1X PBS and into three 384-well plates that were pre-filled with 50 µL of PBS. After completing the sort, the plates were centrifuged at 100×g for 30 s and then imaged on the Celigo (Nexcelom Bioscience LLC, Massachusetts, USA) for fluorescence using the green channel (483/536 ex/em). The Cell Counting application was used to automatically count the number of beads per well. The number of wells containing a single bead was divided by the number of wells on the plate to determine sorting efficiency. The results were reported as mean ± standard deviation (SD). This was performed with three single cell cartridges.

### Cell lines

The cell lines used were CHO ES (Expression Systems, Davis, USA), HEK293 GFP (GenTarget Inc, San Diego, USA), HEK293 GFP-RFP (GenTarget Inc, San Diego, USA), Expi293F (Gibco, USA), CHO-GFP (GenTarget Inc, San Diego, USA), Jurkat Clone E6-1 (ATCC TIB-152), MCF-7 (ATCC HTB-22), and A549 (ATCC CCL-185).

### Growth medium

CHO ES were grown in ESF SFM Mammalian Cell Culture Medium (Expression Systems, Davis, USA). Expi293F were grown in Expi293 Expression Medium (Gibco, USA). Both of these suspension cell lines were grown in 125 mL Erlenmeyer shaker flasks (TriForest Labware, Irvine, USA) without the addition of animal-derived supplements. Adherent cells were grown in T75 tissue-treated culture flasks (Genesee Scientific, San Diego, USA). HEK293 and MCF-7 were grown in Dulbecco’s modified Eagle medium (DMEM) (Genesee Scientific, San Diego, USA). Meanwhile, A549 and CHO-K1 were grown in Ham’s F-12K (Kaighn’s) medium (Gibco, USA). Jurkat is a suspension cell line that was grown in RPMI-1640 (Gibco, USA) in T75 suspension cell flask (CellTreat, Massachusetts, USA). The basal medium used to grow HEK293, MCF-7, A549, CHO-K1, and Jurkat was supplemented with 10% FBS (Genesee Scientific, San Diego, USA) and 1% antibiotic–antimycotic (Invitrogen, USA). The cells were sub-cultured every 2–3 days and kept up to passage 25.

### Sample and sheath buffer preparation

Adherent cells, such as MCF-7, CHO-K1, HEK293, and A549, were sorted using a sheath buffer composed of 1X PBS, 0.5% bovine serum albumin (BSA) (Thermo Scientific, USA), 5 mM EDTA (Invitrogen, USA), and 12.5 mM HEPES (Gibco, USA). For Jurkat, a sheath buffer composed of 1X PBS, 0.5% BSA, and 25 mM HEPES was used. Finally, for the serum-free suspension cell lines, we used the corresponding basal medium they grow in as sheath buffer supplemented with 12.5 mM HEPES. The sheath buffer was also used as sample buffer when preparing the cells for sorting. Buffers were filtered with 0.22 µm syringe filters (CellTreat, Massachusetts, USA) prior to use.

### Cloning medium preparation

Specific cloning media was prepared for the type of cell line used (Table 1). HEK293 and A549 used Fluorobrite DMEM with 10% FBS and 4 mM GlutaMAX (Gibco, USA). Expi293F was grown in HE150 Medium (GMEP Cell Technologies, Japan) that was supplemented with 6 mM GlutaMAX. CHO ES was grown in a cloning medium composed of 20% ESF SFM Expression Medium with 80% EX-Cell CHO medium (Millipore Sigma, Temecula, USA) that was supplemented with 6 mM GlutaMAX, 1X MEM non-essential amino acids (Gibco, USA), and 1:80 ClonaCell CHO ACF Supplement (StemCell Technologies, Vancouver, Canada).

**Table 1:** Preparation of cloning media to optimize single cell outgrowth: better cell outgrowth was obtained when using cloning media that contained nutrients to aid in the survival of cells at a single cell level

Cell line	Cloning medium
CHO K1	Ham’s F-12 K (Kaighn’s) medium, 10% FBS, 1% antibiotic–antimycotic
CHO ES	80% EX-Cell CHO, 20% ESF SFM, 6 mM GlutaMAX, 1X MEM non-essential amino acids, 1:80 ClonaCell-CHO ACF Supplement
Expi293F	HE150 Medium, 6 mM GlutaMAX
HEK293	FluoroBrite DMEM, 10% FBS, 4 mM L-GlutaMAX
MCF-7	DMEM, 10% FBS, 1% antibiotic–antimycotic
Jurkat	RPMI, 10% FBS, 10% conditioned medium
A549	FluoroBrite DMEM, 10% FBS, 4 mM GlutaMAX

MCF-7 cells were grown in DMEM with 10% FBS and 1% antibiotic–antimycotic. CHO-K1 were grown in basal medium composed of Ham’s F-12K (Kaighn’s) medium with 10% FBS and 1% antibiotic–antimycotic. Lastly, Jurkat cells were grown in RPMI-1640 supplemented with 10% FBS and 10% conditioned medium.

### A549 transduction

A549 cells were transduced with pre-made GFP (CMV, Bsd) lentiviral particles (GenTarget, San Diego, USA). Cells were seeded into a 6-well plate and were ready for transduction when the cells were at 70% confluency. On the day of transduction, A549 cells were transduced using a multiplicity of infection (MOI) of 5. After 72 h post-transduction, the cells were prepared in sheath buffer composed of 1X PBS, 0.5% BSA, 5 mM EDTA, and 12.5 mM HEPES. The transduced A549 cells were then sorted using the WOLF to isolate 1 cell per well for cells that were GFP-positive into pre-filled 96-well plates containing 200 µL of optimized cloning medium (Table 1). Additionally, the transduced cells were also sorted using a bulk sort cartridge to determine the transduction rate and post-sort enrichment.

### Single-cell sort

The tested cell lines were sorted at 1 cell/well to determine the single cell efficiency and outgrowth of colonies originating from a single cell. The cells were sorted into 96-well plates containing optimized cloning medium combinations to ensure single cells can survive and grow into well-established colonies. The tested cells were analyzed on the WOLF to acquire a scatter plot and create a gate to exclude debris. This sample gate was then used to create a singlets gate to remove doublets from the sort. Depending on the cell line, additional parameters were used to perform the single cell sort to isolate the cell population of interest.

CHO ES, Expi293F, and Jurkat cells were sorted label-free with the doublets excluded. HEK293, CHO-K1, and A549 cells were sorted based on singlets followed by GFP fluorescence with no viability dye. HEK293 cells that were dual-expressing GFP and RFP were sorted based on being able to express both fluorophores while excluding doublets and without using viability dyes. MCF-7 live cells were sorted based on dead cell exclusion using DRAQ7 (BioStatus, Leicestershire, United Kingdom) as the viability dye.

Prior to sorting, the cells were prepared to have a final concentration of 1.0 × 10^5^ cells/mL in the same buffer used for the sheath and were filtered using 35 µm filter cap tubes (Genesee Scientific, San Diego, USA). Using a sterile single cell cartridge, the cells were sorted into three 96-well plates that were pre-filled with 200 µL of cloning medium specific to each cell line to enhance outgrowth. CHO-K1, CHO ES, HEK293, MCF-7, Jurkat, and A549 were grown in Tissue Culture 96-well plates (CellTreat Scientific Products, USA). While Expi293F were sorted in ultra-low attachment 96-well plates (Crystalgen, Inc, New York, USA).

### Limiting dilution

Additional 96-well plates were prepared by limiting dilution to function as a control to demonstrate that using the WOLF for single cell cloning is much more efficient than using this method. The cells were unsorted samples and were diluted to deposit 0.5 cells/well into 96-well plates. For the CHO-K1 GFP cell line, the sample used for limiting dilution was prepared at a 3:1 ratio with a non-GFP expressing wild type CHO-K1 (ATTC CCL-61) to imitate using this method when attempting to create a new cell line without the ability to isolate the cell population of interest. The cells were also grown on the optimized cloning media for the tested cell line.

### Analysis of cell outgrowth

Plates were maintained in a 37 °C incubator, 5% CO_2_, and analyzed on day 0, day 7, and day 14 using the Celigo. CHO-ES, Jurkat, Expi293F, and MCF-7 cells that did not express a fluorescent protein were analyzed using only the brightfield channel. Meanwhile, CHO-K1 GFP, A549, and HEK293 GFP were analyzed with the brightfield channel alongside the green channel. Finally, HEK293 GFP-RFP were analyzed with the brightfield channel, green channel, and red channel (531/629 ex/em) channel.

For day 0, the Direct Cell Counting application was used on the Celigo to image the number of wells deposited and identify empty wells, wells with a single cell, and wells with 2 or more cells. Analysis was also performed on day 7 and day 14 using the Colony Application. After confirming which colonies originated from a single cell, the data from day 0 and day 14 was saved in heatmaps. Using the Celigo, the number of wells containing a single colony was counted automatically using the Single Colony Verification application. Single-cell origin outgrowth was calculated by counting the number of wells that contained a single colony divided by the number of wells of the culture plate after day 14 and ensuring that the colony originated from a single cell from the cell deposition on Day 0 and comparing the growth to day 7 and day 14. The results of monoclonal outgrowth were represented as mean ± SD in percent to compare outgrowth when using the cell sorter compared to limiting dilution. This experiment was performed in triplicates for each tested cell line.

## Results and discussion

**Fig. 1:**
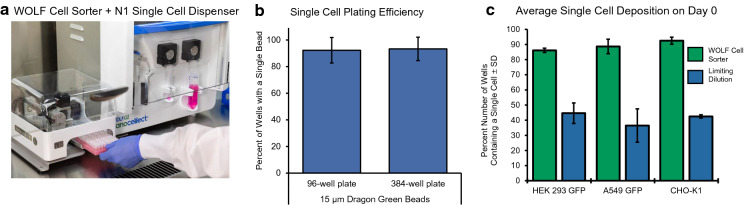
Single bead or cell deposition when using the WOLF and N1 Single Cell Dispenser for sorting 15 µm Dragon Green beads and different GFP-positive cells lines. **a** The WOLF Cell Sorter and the N1 Single Cell Dispenser easily can fit in the hood to maintain sterility while sorting cells due to being compact. **b** The ability to deposit a single bead per well resulted in an average dispense efficiency of 92.3 ± 9.6% when sorting beads into 96-well and 93.3 ± 8.8% when sorting into 384-well plates. **c** When sorting GFP-positive cells lines, there was an average single cell deposition on day 0 of 86.1% for HEK293 GFP, 88.7% for A549, and 92.5% for the CHO-K1. This in comparison with limiting dilution where the single cell deposition was 44.6% for HEK293, 36.5% for A549, and 42.5% for CHO-K1

As previously mentioned, SCC cloning is required for numerous applications, such as therapeutic protein production, drug screening, gene therapy, and understanding cell function and disease mechanisms. Single cell cloning was performed in a tissue culture hood to maintain sterility when sorting the cells (Fig. 1a). Single cell deposition using the WOLF Cell Sorter was first validated with 15 µm Dragon Green Beads. The control beads are very bright and easy to visualize allowing them to be useful for counting when using a plate imager. Single bead sorting of 15 µm Dragon Green beads into 96-well and 384-well plates resulted in a sorting efficiency of 92.3 ± 9.6% and 93.3 ± 8.8%, respectively (Fig. 1b).

Validating single-cell deposition of cells is more difficult at day 0 and can sometimes be incorrectly classified. Cell dyes can be used but is not desired due to their potential toxicity and effect on cell growth. Here we used multiple cell lines expressing GFP to assist in better classifying single cells in a well at day 0. We utilized three common cell lines (Chinese hamster ovary (CHO) cells, HEK293 cells, and A549 cells) that were engineered to have high GFP expression to assist in classifying single cells per well at day zero. The CHO-K1 cell line, which was originally derived as a subclone from the parental CHO cell line, has been used in culture for more than 50 years to produce therapeutic proteins such as ENBREL (a TNF inhibitor) and HERCEPTIN (an anti-HEK-2 breast cancer antibody) (Wurm 2013). CHO K1-GFP cells were sorted and dispensed in the same way as the control Dragon Green Beads and demonstrated similar single-cell plating efficiency of 90.8 ± 4.3% on day 0. Meanwhile, limiting dilution showed an efficiency of 42.5 ± 1.1% (Fig. 1c). Two additional cell lines were also tested, which were human embryonic kidney cell line HEK293 and A549. HEK293 cells are used to create a variety of therapeutics and the human adenocarcinoma cell line A549 has been used as a lung cancer cell line for drug screening (Swain et al. 2010; Dumont et al. 2016). HEK293 GFP and A549 GFP cells were sorted into 96-well plates and were measured at day 0 resulting on an average single cell depositions of 86.1 ± 1.4% and 88.7 ± 4.8%, respectively. Sorting of the three distinct cell lines demonstrated considerably higher single-cell deposition compared to limiting dilution which had an average single-cell efficiency of only 44.6 ± 6.8% for HEK293 GFP and 36.5 ± 6.5% for A549 on day 0 (Fig. 1c).

**Fig. 2:**
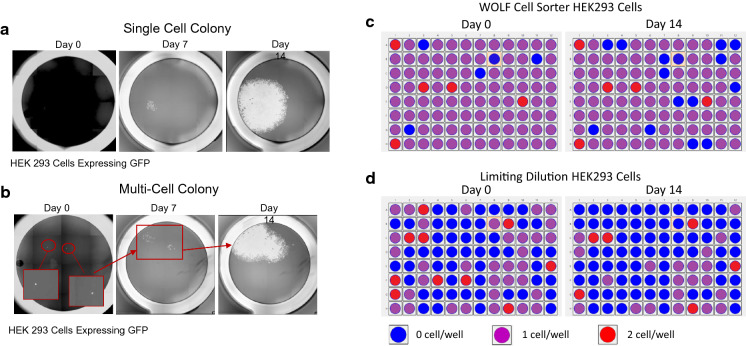
Representative images of the analysis for a GFP-positive cell line. **a** Day 0 analysis was used to determine if the colony on Day 7 and Day 14 originated from a single cell or **b** from multiple cells per well. On Day 14, the heatmaps for the plates prepared with **c** the WOLF Cell Sorter or with **d** limiting dilution were saved to calculate the number of wells with outgrowth originating from a single cells. Wells containing a single cell were highlighted in purple. Meanwhile, empty wells were highlighted in blue and wells containing two cells or more were denoted with red

In order to demonstrate the cell’s ability to grow into healthy colonies, it is important to grow single-cell clones for up to 14 days and monitor the cells over time. Single-cells visualized at day zero can be fully confirmed by day 7 when a small and round cluster of cells is detected near the same area where the single cell was first observed. A single large and round cluster of viable cells observed by day 14 is a further indication that the colony has a single cell origin and growing well (Fig. 2a). Single clusters that appear “pear shaped” by day 14 are usually the result of two separate cells dispensed at day zero (Fig. 2b).

A significant drop in viable colonies of cells from day 0 to 14 may indicate that the sorted cells were not healthy prior to sorting, damaged during the sorting process, the culture media conditions are not optimal for growth, or a combination of those three. We compared the number of wells having HEK293 GFP colonies with single-cell origin after sorting on day 0 versus day 14 and observed an average of 86.1 ± 1.4% single-cell colonies on day 0 and 73.7 ± 3.3% by day 14, which was a 14% drop in viable single-cell colonies (Fig. 2c). However, when performing limiting dilution, we overserved only an average of 44.6 ± 6.8% single-cell colonies on day 0 and 18.9 ± 6.5% by day 14, which was a 58% drop in viable single-cell clones (Fig. 2d).

**Fig. 3:**
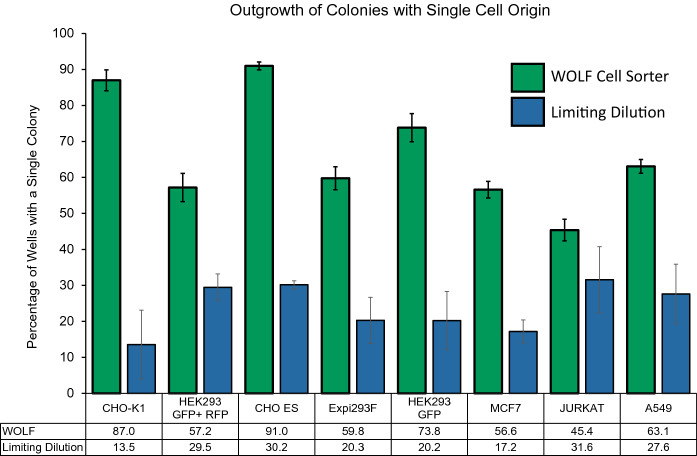
Percentage of Wells Containing One Colony per Well on Day 14. Using the WOLF for cell line development resulted in an average of 66.7 ± 15.9% across the tested cell lines compared to an average of 23.8 ± 6.8% when using limiting dilution across all tested cell lines

To demonstrate SCC across a wider variety of cell types we measured outgrowth of additional cell lines. We tested an engineered HEK293 cell line having both GFP and RFP expression to compare outgrowth between cell lines expressing single and dual-fluorescent proteins. We tested suspension CHO-ES cells to compare against adherent CHO-K1 cells. Lastly, we also tested Jurkat cells which were isolated in 1977 and have become an in vitro system useful to study T-cell biology and MCF-7 which is a breast cancer cell line that was established in 1973 and has been used to study estrogen responses, hormone resistance, and testing of potential cancer therapeutics such as tamoxifen (Cassioli et al. 2021; Lee et al. 2015). Additionally, MCF-7 has been used to investigate the importance of the interaction between cancer cells, angiogenesis, cellular metabolism, and respiration (Comşa et al. 2015). By day 14, all the tested cell lines demonstrated significantly higher single-cell outgrowth compared to limiting dilution. Altogether, the comprehensive variety of cell lines tested is summarized in Fig. 3 and highlights the utility and overall increased productivity that can result from using the WOLF Cell Sorter compared to limiting dilution.

Using a microfluidic cell sorter was advantageous in SCC due to being able to select and deposit more single cells per well compared to limiting dilution. Furthermore, limiting dilution will require three to four subcloning steps since colonies with single cell origin cannot be guaranteed regardless of the statistics behind limiting dilution. This method suffers from a phenomenon known as persistence of mixed clones where mixed clones can remain even after many cycles of re-cloning (Kuystermans and Al-Rubeai 2014).

The importance of media optimization was observed during preliminary experiments. For example, initial testing for the outgrowth of suspension CHO ES cells used basal growth medium instead of the optimized cloning medium.

These initials tests resulted in no cells growing in the sorted 96-well plates nor in the plate prepared via limiting dilution (data not shown). However, once the cloning medium was supplemented with the formulation described in Table 1 for CHO ES, the total outgrowth increased. Another initial test consisted of growing A549 with basal growth medium, which resulted in an outgrowth of 50.0 ± 4.54%. Once the cloning medium was changed to DMEM, the outgrowth increased to 63.1 ± 5.7%. Cell lines that were prone to undergo rapid cell death, such as MCF-7, benefited from using a viability dye to exclude dead cells from the sort. MCF-7 had monoclonal outgrowth of 56.6 ± 2.3% when using a viability dye compared to 17.2 ± 3.2% when using limiting dilution where a viability dye cannot be used to select for the live cells only.

### Gating cells for improved selection of desired single cells

**Fig. 4:**
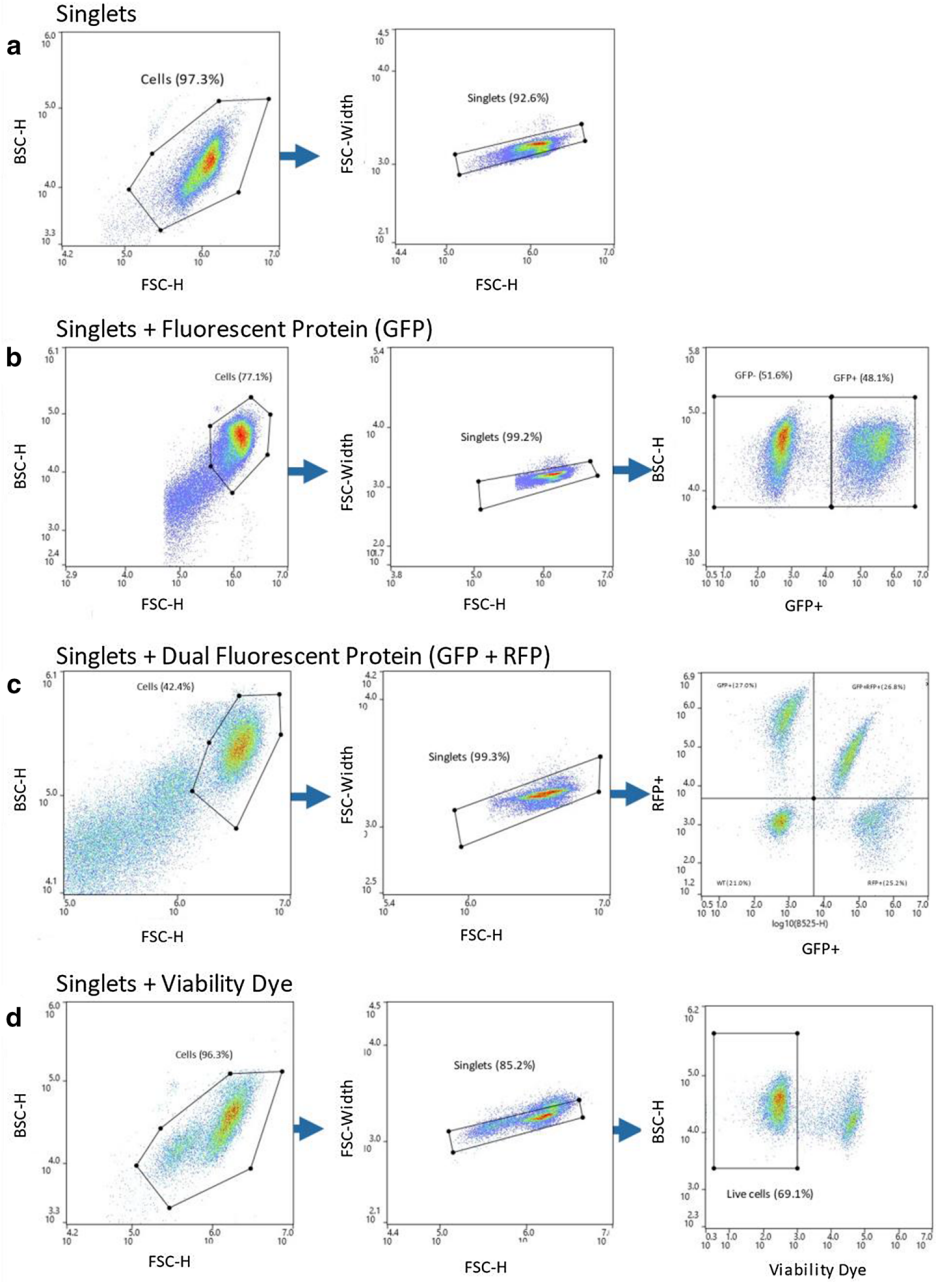
Gating Strategies for Specific Single Cell Isolation. The population of interest were gated on the plots to ensure that the target cell of interest is isolated during the sort while removing debris and doublets. **a** Gating strategy for cells without the need of any dyes was used to sort CHO ES, Jurkat, and Expi293F. Gating strategy for the cells that expressed fluorescence proteins such as **b** only GFP or **c** cells that dual-express GFP-RFP. **d** Gating strategy for cells that were stained with a viability dye

Sorting of single-cells into wells based on specific gates provides the ability to select cells using a combination of parameters to improve the selection process. The simplest gate to perform is to gate for singlets based on scatter properties (Fig. 4a). Gating for singlets does not rely on dyes or fluorescent proteins and decreases the probability of dispensing two or more cells close together. If the cell line is engineered to express bright fluorescent proteins like GFP or RFP, then the expression levels can be used to sort singlets only expressing GFP (Fig. 4b), RFP or both GFP and RFP together (Fig. 4c). If viability dyes are acceptable for a particular assay, then addition of propidium iodine (PI) or other viability dyes can be used to dispense only singlet live cells (Fig. 4d).

**Fig. 5:**
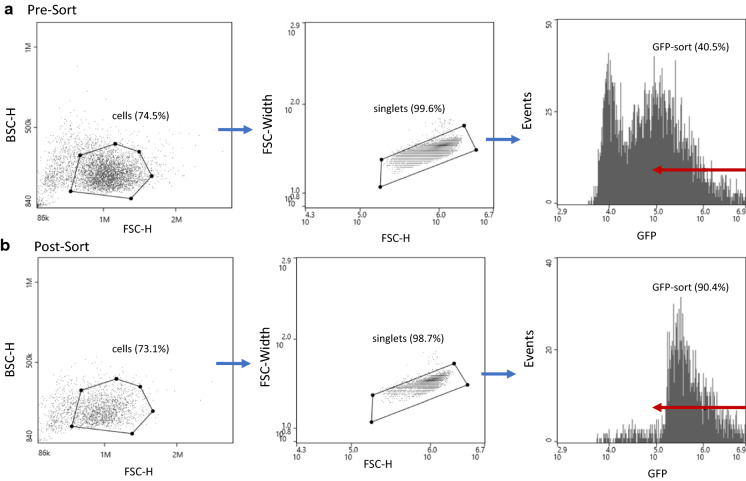
Sorting of Transduced A549 after using an MOI of 5. The scatter plot was used to exclude debris followed by a singlets gate to remove doublets was applied to a GFP-positive histogram. **a** The sort gate (labeled in red) was used to sort the cells that expressed the highest levels of GFP, which before sorting was composed of a target population of 40.5%. **b** After completing the sort, the sample was purified up to 90.4%

To highlight the importance of gating, we transduced A549 cells that can express GFP at varying levels. When using an MOI of 5, the transduction resulted in 69.5% of the cells expressing GFP (Fig. 5a). For simplicity, we performed bulk sorting to gate and isolate cells that were the top 40.5% GFP expressors. After sorting, the high A549 cells that retained the high GFP expression was 90.4% (Fig. 5b). The same gate used for the bulk sort was used to sort A549 cells for single cell cloning. This resulted in a monoclonal outgrowth of 63.1 ± 5.7% compared to limiting dilution where 27.6 ± 3.7% of the colonies came from a single cell. Altogether, these simple gating strategies can greatly increase the probability for sorting and dispensing healthy, single cell clones across a 96 or 384-well plate.

## Conclusion

In this work, we demonstrate how the use of the WOLF Cell Sorter alongside the N1 Single Cell Dispenser can be an ideal platform to increase the number of single-cell origin colonies due to combining the ability to select specific cells of interest and depositing one cell per well. Using optimized cloning media to ensure single cell survival was also an important step to ensure that the cells continue growing for further downstream applications. The capability of selecting for important clones is lacking when using limiting dilution, which results in the increase of time needed to isolate the clone of interest.
